# Multi-Injection Pharmacokinetics of Meloxicam in Kemp’s Ridley *(Lepidochelys kempii*) and Green (*Chelonia mydas*) Sea Turtles after Subcutaneous Administration

**DOI:** 10.3390/ani11123522

**Published:** 2021-12-10

**Authors:** Terry M. Norton, Tonya Clauss, Rachel Overmeyer, Stephanie Stowell, Michelle Kaylor, Sherry Cox

**Affiliations:** 1 Department of Rehabilitation and Veterinary Services, Georgia Sea Turtle Center/Jekyll Island Authority, 214 Stable Road, Jekyll Island, GA 31527, USA; rovermeyer@jekyllisland.com (R.O.); sstowell@jekyllisland.com (S.S.); mkaylor@jekyllisland.com (M.K.); 2 Georgia Aquarium, 225 Baker Street NW, Atlanta, GA 30313, USA; tclauss@georgiaaquarium.org; 3 Department of Environmental and Diagnostic Sciences, College of Veterinary Medicine, University of Tennessee, Knoxville, TN 37996, USA; scox6@utk.edu

**Keywords:** Kemp’s ridley sea turtle, green sea turtle, meloxicam, pharmacokinetic, multi-injection

## Abstract

**Simple Summary:**

Drug dosing in sea turtles is often extrapolated from other reptiles, mammals, and humans. A more accurate way to determine the appropriate dose of a particular drug is by performing pharmacokinetic studies in that species. Meloxicam is an anti-inflammatory and pain management drug commonly used in humans and a wide range of animals including sea turtles. The authors recently published a study on single-injection meloxicam pharmacokinetics in sea turtles. The current study is a continuation of the single-injection study with objectives to determine the appropriate frequency of meloxicam administration in Kemp’s ridley and green sea turtles. Further, we evaluated whether the drug accumulated in the blood after multiple injections and if it caused any side effects. The results show that Kemp’s ridley sea turtles should receive a dose of 1 mg/kg subcutaneously every 12 h, whereas in green turtles, this same dose should be used at a frequency of every 48 h. No adverse side effects or statistically or clinically significant changes to blood work parameters were noted. This study provides important information to enhance pain management in endangered sea turtles undergoing rehabilitation.

**Abstract:**

The objective of this study was to determine the pharmacokinetics and safety of multiple injections of meloxicam (MLX) administered subcutaneously (SQ) in Kemp’s ridley (*Lepidochelys kempii*) and green (*Chelonia mydas*) sea turtles. Based on results from a previously published single-injection study, a multiple-injection regimen was derived for the Kemp’s ridleys, which consisted of administering MLX at a dose of 1 mg/kg SQ every 12 h for 5 days, and for green turtles at a dose of 1 mg/kg SQ every 48 h for three treatments. Six turtles of each species were used for the study, and blood samples were taken at multiple time intervals. The terminal half-life after the last dose for the Kemp’s ridley sea turtles was calculated at 7.18 h, and for the green sea turtles at 23.71 h. Throughout the multiple injections, MLX concentrations remained above 0.57 µg/mL, a concentration targeted in humans for the analgesic and anti-inflammatory effects. No negative side effects or changes to blood parameters evaluated were observed during the study in either species. The results of this study suggest MLX should be administered SQ to Kemp’s ridley sea turtles at a dosage of 1 mg/kg every 12 h and in green sea turtles at a dose of 1 mg/kg every 48 h. The novelty of this work is that it is a multiple-injection study. Multiple injections were administered and produced concentrations that were considered therapeutic in humans, and the turtles did not have any adverse side effects. Furthermore, there were large differences in the pharmacokinetic values between green and Kemp’s ridley sea turtles.

## 1. Introduction

In sea turtle rehabilitation, traumatic injuries such as limb amputations and shell and long bone fractures often occur secondary to boat strikes, predator attacks, and entanglement in fishing gear and often require surgery [1,2]. Fibropapillomatosis is common in green turtles presenting to rehabilitation centers and is often managed by CO_2_ laser tumor removal, which produces significant postoperative pain [3]. Significant information has been published on the capacity of reptiles to perceive pain from these types of injuries and surgical procedures [4,5]; thus, appropriate perioperative and postsurgical pain management in these cases is critical [1,2,3]. A reduced time to return to feeding has been reported in sea turtles after receiving analgesic drugs [6]. Until recently, pharmacokinetic data on analgesic drugs in reptiles including sea turtles were very limited, meaning dosage regimens were generally extrapolated from other animal species. Extrapolating drug dosages from mammals, birds, reptiles, or even different species of turtles to sea turtles may result in clinical failure or toxicity. Reptiles have a wide range of unusual behavioral, physiologic, and anatomic characteristics that may affect drug metabolism. Some recent studies are beginning to unravel the complexities of pain management in reptiles including sea turtles [7,8,9,10,11,12,13,14,15,16,17].

Meloxicam (MLX) is used for pain management and to reduce inflammation in a variety of species [18]. It is a cyclooxygenase-2 selective non-steroidal anti-inflammatory drug (NSAID) that is metabolized by the liver, and the inactive metabolites are eventually excreted in the feces and urine [18]. Meloxicam pharmacokinetic studies have been performed in ball pythons (*Python regius*) [19], green iguanas (*Iguana iguana*) [10], and loggerhead sea turtles (*Caretta caretta*) [8,12]. A dose of 0.1 mg/kg MLX was administered intramuscular (IM) and intravenous (IV), and orally in both the loggerhead studies; plasma MLX levels considered to provide analgesia (0.57 µg/mL) in humans were not achieved, and measurable plasma levels were maintained for only a few hours. Similar results were found in red-eared sliders administered a dose of MLX at 0.2 mg/kg [9]. Anti-inflammatory effects of plasma MLX concentrations have been shown to be significantly variable among mammalian species studied. In humans, the range is 0.57 to 0.93 µg/mL [20], while in horses and dogs, the range is 0.13 to 0.19 µg/mL and 0.82 µg/mL, respectively [21,22]. Efficacy and pharmacodynamic studies for MLX have not been conducted in any turtle species; thus, in this study, we used 0.57 µg/mL as the plasma drug target level.

A study was recently published by the authors on single-injection MLX pharmacokinetics in three species of sea turtles [14]. In Kemp’s ridley and green turtles, a dose of 1 mg/kg MLX was adminstered SQ. The half-life (T1/2) of MLX was 5.51 h in the Kemp’s ridleys but could not be determined in the greens. After a dose of 1 mg/kg of MLX administered SQ in Kemp’s ridley and green sea turtles, the maximum concentration (C_max_) for MLX was 6.76 µg/mL and 9.35 µg/mL, respectively. Measurable plasma concentrations in Kemp’s ridley sea turtles occurred for 48 h, while in green sea turtles, MLX was detected for 120 h. No adverse side effects were noted. In loggerhead sea turtles, the half-life of MLX administered SQ at a dose of 2 mg/kg was 2.99 h. The C_max_ was 3.63 µg/mL, and measurable plasma levels only lasted for 24 h. Based on this study, a dose of 1 mg/kg of MLX admintered SQ to Kemp’s ridley and green turtles produced plasma concentrations greater than 0.57 µg/mL for 12 h and 120 h, respectively. In loggerhead sea turtles, plasma concentrations of MLX above 0.57 µg/mL were only maintained for 4 h, even at a higher dose of MLX of 2 mg/kg SQ. One of the authors (TMN) has found that MLX anecdotally shortens the return to feeding in green turtles after surgical removal of fibropapilloma tumors with CO_2_ laser surgery.

The objective of this study was to establish the pharmacokinetics and safety of MLX after multiple SQ injections in Kemp’s ridley and green sea turtles. Because therapeutic levels in loggerheads were maintained for only a short period of time in the single-injection study, this species was not included in this study. Studies such as this are extremely valuable in understanding the appropriate dose and frequency of a drug. After single-injection drug administration, the plasma drug level rises above and then falls below the minimum effective concentration (MEC), resulting in a decline in the therapeutic effect. To maintain prolonged therapeutic activity, many drugs are administered in a multiple-injection regimen. The plasma levels of drugs administered in multiple injections must be maintained within the limits of the therapeutic window to achieve optimal clinical effectiveness. While single-injection pharmacokinetic studies provide a glance into the basic characteristics of drug deposition, multiple-injection pharmacokinetic studies are necessary to characterize the drug disposition in a manner that is consistent with its intended clinical use. Single-injection pharmacokinetic study parameters provide information for predicting the average concentration at steady state, if linear and time-invariant pharmacokinetics applies. However, these assumptions are commonly violated when the drug hepatic metabolism is saturated or when the drug clearance and volume of distribution change slightly with time, a situation that is common with metabolically eliminated drugs. Therefore, repeated administration studies are necessary to understand the relation between dose and the drug concentration profile at steady state and to confirm the predictions made from single-administration pharmacokinetic parameters.

## 2. Materials and Methods

Six juvenile Kemp’s ridley and six juvenile green sea turtles were used in this study. All turtles were being rehabilitated at the Georgia Sea Turtle Center (GSTC) on Jekyll Island, Georgia. The health of the turtles was evaluated by conducting a physical examination and blood work which included complete blood counts, plasma biochemical panels, and protein electrophoresis (PEP). A small amount of heparinized whole blood was transferred to a microhematocrit tube and centrifuged to measure packed cell volume. Plasma total solids were measured by a refractometer. White blood cell estimates and differential counts were performed by the same person. Biochemistry profiles were performed on plasma samples using standard dry-slide determinations with a Kodak 700XRTM chemical analyzer by the Department of Pathology, University of Miami (Miami, FL, USA). Vitros Performance Verifiers I and II (Ortho Diagnostics, Rochester, NY, USA) were used to test the chemistry analyzer. Results from the solutions, representing high and low controls, were compared to Vitros ranges. The analyzer was also calibrated with Ortho Vitros reagents. Any ‘‘flags’’ or errors were fully investigated. The following blood values were measured: glucose, sodium, potassium, carbon dioxide, blood urea nitrogen (BUN), creatinine, BUN/creatinine ratio, phosphorus, calcium, uric acid, creatine phosphokinase (CPK), alanine aminotransferase (ALT), aspartate aminotransferase (AST), lactate dehydrogenase (LDH), lipase, amylase, cholesterol, glucose, and gamma-glutamyl transferase (GGT). Protein fractions were evaluated by plasma electrophoresis. Blood analyte data were reported as median (minimum, maximum). The blood parameters were tested by using the non-parametric Wilcoxon signed rank test. Values of *p* ≤ 0.05 were considered significant.

All turtles used in the study were eating normally. The weight and straight carapace length of the Kemp’s ridley sea turtles ranged from 3.15 to 5.5 kg and 27.20 to 34.10 cm, and those of the greens ranged from 3.0 to 7.30 kg and 28.40 to 38.70 cm. All turtles used in the study were either considered to be a permanent captive or were nearing release. The sea turtles were housed in circular fiberglass tanks measuring 8 feet (2.43 m) × 8 feet (2.43 m) or 10 feet (3 m) × 10 feet (3 m). Salt water was maintained at a temperature between 74 and 77 °F (23 to 25 °C). A closed filtration system was used which included a protein skimmer, biological filter, bead filter, and ozone for disinfection. A diet of fish, squid, and crabs with multivitamin and calcium supplementation was fed to the Kemp’s ridley sea turtles. Green sea turtles were fed a variety of leafy greens, an in-house prepared gel diet, and multivitamin and calcium supplementation [14].

Baseline MLX plasma levels were measured 2 weeks prior to MLX administration. All turtles received MLX (injectable 5 mg/mL, Putney Inc., Portland, ME, USA) SQ in the shoulder region. Injection sites were changed from right to left to avoid local reactions. For the Kemp’s ridleys, MLX was administered at a dose of 1 mg/kg SQ every 12 h for 5 days for a total of 10 injections. A volume of 0.3 mL of heparinized blood was collected from the external jugular (cervical sinus) at time 0, then the first injection of MLX was administered, then 0.3 mL of blood was collected at 12 h, and then next injection of MLX was administered at 12 h. MLX was administered SQ every 12 h for 10 treatments with a blood sample taken prior to each treatment. Blood was collected at 5, 15, 30, and 45 min, and 1, 4, 8, 12, 24, 48, and 72 h after the last injection of MLX was administered (96 h). The total blood volume taken for the 5-day period was 6.9 mL. Green turtles were administered 3 injections of MLX at 1 mg/kg SQ every 48 h. Sample collection volume was 0.3 mL for each time with collection at time 0, 48, and 96 h, and then after the 96 h injection, blood was collected at 5, 15, 30, and 45 min, and 1, 4, 8, 12, 24, 48, 72, 96, 120, and 144 h. The total volume of blood obtained was 5.1 mL over 6 days. A complete blood count, plasma chemistry panel, and protein electrophoresis were performed approximately 1 week after the study was completed to assess general health and, more specifically, kidney and liver function. Shortly after collection, the blood was centrifuged, and plasma was then placed in a cryovial and frozen at −80 °C until plasma MLX analysis took place. Dry ice was used to ship the plasma samples to the University of Tennessee College of Veterinary Medicine.

The animal care staff at the GSTC evaluated each turtle after they received the injection for any changes in swimming, intensity of flipper movement, posture in the water, mentation, respiratory rate, appetite, activity level, and any other changes in behavior. Additionally, the turtles were carefully monitored for a four-day period after injections were completed. The injection site was examined immediately post-injection and prior to the next injection for any redness or swelling.

A validated high-performance liquid chromatography (HPLC) system was used to measure plasma MLX levels [23]. The chromatography system was composed of a 2695 separations module and a 2487 ultraviolet detector (Waters, Milford, MA, USA). MLX was separated on an Xbridge C_18_ column (Waters, Milford, MA, USA) with a flow rate of 1 mL/min. The mobile phase was composed of glacial acid in water (pH0 and acetonitrile (50:50, *v/v*), and absorbance was measured at 360 nm. MLX samples were thawed, and 100 µL of plasma was transferred to a screw-top tube, followed by 15 µL of prioxicam (internal standard, 5 µg/mL), 100 µL of 1 M HCL, and 2 mL of chloroform. The tubes were vortexed for 60 s and underwent centrifugation for 20 min at 1040× *g*. The organic phase was removed and evaporated to dryness with nitrogen. Samples were reconstituted in 250 µL of mobile phase, and 100 µL was injected into the HPLC system. Untreated plasma taken from the turtles in this study was fortified with MLX to prepare standardized curves for plasma analysis. A linear concentration was produced ranging from 5 to 10,000 ng/mL. Calibration samples were also prepared the same way. An amount of 5 ng/mL was the lower limit of quantification. The average recovery of MLX was 95%, and the intra- and inter-assay variability ranged between 1.1 and 10%.

Phoenix WinNonlin 7.0 (Certara Inc., Princeton, NJ, USA) was used for noncompartmental analysis of the MLX data. Area under the plasma concentration time curve (AUC_0-∞_) from time 0 to infinity, maximum plasma concentration (C_max_), elimination rate constant (λ_z_), plasma half-life (t½), and time to maximum plasma concentration (T_max_) were the pharmacokinetic parameters determined. The parameter values were expressed as the mean of the individually estimated parameters, and the variability was reported as the standard deviation, except for the half-life, which was reported as the harmonic mean, and pseudostandard deviation.

The pharmacokinetic parameters from this study were statistically compared to those from the single-injection study by the paired Student *t*-test. Pharmacokinetic parameters from green and Kemp’s ridley sea turtles in the multi-injection study were also compared. Data were analyzed by use of the skewness test to determine normality. All statistical analyses were performed by using Graphpad Prism (San Diego, CA, USA). Mean values were considered significantly different at *p* < 0.05.

## 3. Results

**Figure 1:**
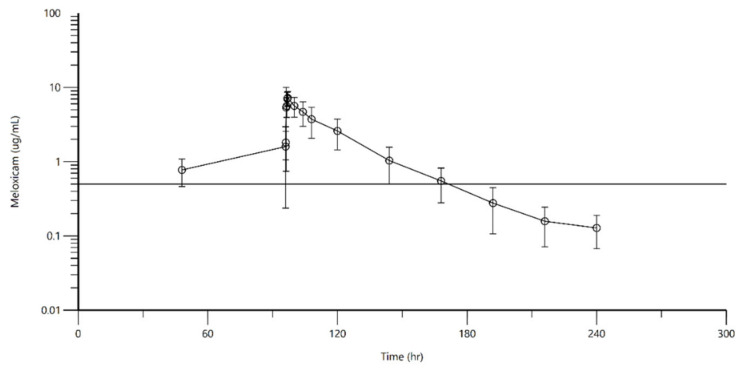
Mean plasma concentration of MLX over time following administration of 1 mg/kg SQ Q48 h for 3 doses (T = 0, 48, 96 h) to 6 green turtles. The reference line represents 0.5 µg/mL.

**Figure 2:**
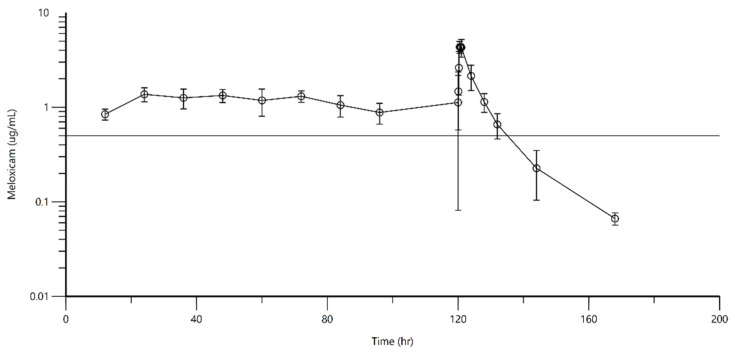
Mean plasma concentration of meloxicam over time following administration of 1 mg/kg SQ Q12 h for 5 days to 6 Kemp’s ridley turtles. The reference line represents 0.5 µg/mL.

No negative side effects were observed during the study in either species. Pre- and post-drug administration behavioral observations by experienced husbandry staff revealed no adverse behavioral or appetite changes in any turtle. Complete blood counts and plasma chemistry profiles were found to be within normal limits prior to the study and approximately 1 week after the study was completed. The null hypotheses showed that blood analytes (paired data) were not significantly different before and after MLX treatment (Supplementary Tables S1–S3). After the last dose for the Kemps, the terminal half-life, maximum concentration, and time to maximum concentration were 7.18 ± 2.21 h, 4.77 ± 0.26 µg/mL, and 0.75 ± 0.27 h, respectively. For the greens, the terminal half-life, maximum concentration, and time to maximum concentration were 23.71 ± 2.81 h, 9.03 ± 2.59 µg/mL, and 1.29 ± 1.35 h, respectively; additional pharmacokinetic parameters are listed in Table 1. There were no statistical differences in any of the pharmacokinetic parameters for either group of turtles. The concentration–time curve for the Kemps is shown in Figure 1, and that of the greens in Figure 2. Throughout the multiple dosing, MLX concentrations remained above 0.57 µg/mL, a concentration targeted in humans for the analgesic and anti-inflammatory effects of the drug.

## 4. Discussion

There are very limited publications on single- and multi-injection analgesic drug pharmacokinetics in reptiles including sea turtles. The purpose of this study was to determine whether multiple injections of MLX administered SQ to Kemp’s ridley and green sea turtles would lead to and maintain blood concentrations over the course of a typical therapeutic regimen consistent with those producing analgesia and anti-inflammatory effects in mammals. An additional goal was to ensure that MLX did not accumulate and reach plasma levels over multiple injections that would lead to adverse side effects. There were no significant changes to behavior, appetite, or blood parameters that were monitored during the course of the study. Furthermore, there were no injections site reactions observed during the course of the study. The dose, route, and frequency of administration for this study were based on a previously published study by the authors on single SQ injection of MLX in Kemp’s ridleys, green, and loggerhead sea turtles [14]. Based on the results from that study, it was determined that MLX does not reach therapeutic levels in loggerhead turtles even after doubling the dose (2 mg/kg); thus, this species was excluded from this study. A dose of MLX of 1 mg/kg administered SQ every 12 h was used in Kemp’s ridleys, and a dose of MLX at 1 mg/kg was administered SQ every 48 h in green turtles. Significant variation exists among mammalian species that have been studied for the concentration of MLX that causes the anti-inflammatory effects, ranging from 0.57 to 0.93 µg/mL in humans [20], 0.13 to 0.19 µg/mL in horses [21], and 0.82 µg/mL in dogs [22]. There have been no MLX efficacy or pharmacodynamic studies conducted in any turtle species; thus, we chose plasma levels that have been determined to be anti-inflammatory in humans for the target levels in this study. The results from this study suggest that administration of MLX SQ at 1 mg/kg every 12 h in Kemp’s ridleys maintained MLX plasma levels above 0.57 µg/mL prior to the 12, 24, 36, 48, 60, and 72 h time periods. Plasma levels were monitored at 5, 15, 30, and 45 min, and 1, 4, 8, 12, 24, 48, and 72 h, after the last dose (96 h). The peak concentration was 4.77 ± 0.26 µg/mL and remained above therapeutic levels for the next 12 h. For green turtles, plasma levels were maintained above therapeutic levels prior to the 48 (second) and 96 (third) h doses. Plasma levels were monitored at 5, 15, 30, and 45 min, and 1, 4, 8, 12, 24, 48, 72, 96, 120, and 144 h, after the 96 h dose. The peak concentration was 9.03 ± 2.59 µg/mL, and concentrations were maintained above therapeutic levels for 72 h after the last dose. There was no accumulation of the drug, behavioral or appetite changes in the turtles, or clinical pathology alterations after multiple injections in either species. The multiple-injection PK parameters calculated (Table 1) were fairly consistent with our previous single-injection study. There were no statistical differences when the parameters were compared. In the green turtles, terminal half-life was not determined in the single-injection study, but in this study, it was calculated to be 23.71 ± 2.81. There were large differences in the pharmacokinetic parameters between the green and Kemp’s ridley sea turtles. The terminal half-life (h) in green turtles was 23.71 ± 2.81, whereas in Kemp’s ridley turtles, it was 7.18 ± 2.21, indicating the pharmacological activity of MLX lasts much longer in green turtles. The elimination rate constant K (1/h) was 0.03 ± 0.003 in green turtles and 0.10 ± 0.03 in Kemp’s ridley turtles, indicating the drug was eliminated more rapidly in Kemp’s ridleys than green turtles. C_max_ (µg/mL) was 9.03 ± 2.59 in green turtles and 4.77 ± 0.26 in Kemp’s ridley turtles, indicating MLX is maintained at a higher plasma concentration between doses in green turtles when compared to Kemp’s ridleys between doses. There were large differences in the values between green and Kemp’s ridley sea turtles for all pharmacokinetic values.

Green sea turtles are herbivorous, feeding primarily on sea grass and algae and utilizing colonic microbial fermentation for digestion. The Kemp’s ridley, on the other hand, is carnivorous, feeding primarily on invertebrate prey. These dietary differences likely affect drug metabolism and pharmacokinetics [24]. The age classes of the two species in this study were similar. Gender was not determined for the turtles in this study, but the differences in pharmocokinetic parameters were so great that it is unlikely to be soley due to gender differences.

## 5. Conclusions

This study confirms that multiple SQ injections of MLX maintain plasma concentrations that are analgesic in mammals and safe for use in Kemp’s ridley and green sea turtles for the endpoints analyzed. The clinical impression of the authors suggests beneficial effects from this course of treatment, as assessed by return to feeding and improved behavior in sea turtles post-surgery after removal of fibropapilloma tumors with a CO_2_ laser. Controlled efficacy studies are needed to assess the analgesic effects of this drug more rigorously in sea turtles. In order to determine the appropriate therapeutic concentration, pharmacodynamic studies need to be performed in each species of sea turtle. It is common practice for veterinarians and rehabilitators to extrapolate drug doses from one sea turtle species to another; however, based on the results of this study, this may not provide accurate dosing and could lead to treatment failures. In future pharmacokinetic studies in sea turtles, the authors recommend evaluating multiple species of sea turtles.

## Figures and Tables

**Table 1:** Pharmacokinetic parameters (mean ± SD) in green turtles following a single SQ administration of 1 mg/kg MLX (*n* = 8) and SQ administration every 48 h for 3 treatments (*n* = 6). Kemp’s ridley turtles following a single SQ administration of 1 mg/kg MLX (*n* = 8) and SQ administration for 5 days, Q12 h (*n* = 6).

Pharmacokinetic Parameter	Green MLX ^a^ Mean ± SD	Green MLX ^b^ Mean ± SD	Kemp MLX ^a^ Mean ± SD	Kemp MLX ^c^ Mean ± SD
Terminal half-life * (h)	^†^	23.71 ± 2.81	5.51 ± 1.58	7.18 ± 2.21
Elimination rate constant λz (1/h)	0.02 ± 0.01	0.03 ± 0.003	0.13 ± 0.04	0.10 ± 0.03
T_max_ (h)	0.81 ± 0.26	1.29 ± 1.35	0.57 ± 0.31	0.75 ± 0.27
C_max_ (µg/mL)	9.35 ± 1.61	9.03 ± 2.59 ^‡^	6.76 ± 3.13	4.77 ± 0.26
AUC_0–∞_ (h∙µg/mL)	286.10 ± 212.71	185.97 ± 70.69 ^‡^	35.55 ± 11.56	32.86 ± 7.70
MRT_0–∞_(h)	50.43 ± 29.32	31.29 ± 3.94 ^‡^	7.21 ± 1.96	9.73 ± 1.27

^a^ Single dose (1 mg/kg SQ), ^b^ multidose (1 mg/kg SQ Q48 h for 3 treatments), ^c^ multidose (1 mg/kg SQ Q12 h for 5 days), * harmonic mean, elimination rate constant (λ_z_), terminal half-life (t½), maximum plasma concentration (C_max_), time to maximum plasma concentration (T_max_), area under the plasma concentration time curve from time 0 to infinity (AUC_0–__∞_), mean residence time (MRT_0–__∞_). ^†^ Not enough points to calculate an accurate half-life value. ^‡^ Value is statistically different than that of Kemps after multiple administrations (*p* < 0.05).

## Supplementary Materials

The following are available online at www.mdpi.com/article/10.3390/ani11123522/s1, Table S1: Pre- and post-MLX treatment blood analytes in green and Kemp’s ridley sea turtles, Table S2: Pre- and post-MLX treatment blood analytes in green sea turtles, Table S3: Pre- and post-MLX treatment blood analytes in Kemp’s ridley sea turtles.
