# Supplementary material for: Multi-Injection Pharmacokinetics of Meloxicam in Kemp’s Ridley (Lepidochelys kempii) and Green (Chelonia mydas) Sea Turtles after Subcutaneous Administration
Source: Animals (Basel). 2021 Dec 10;11(12):3522. doi: 10.3390/ani11123522 (PMC8698120; doi:10.3390/ani11123522)
Supplement: Supplementary file 1 [file animals-11-03522-s001.zip › animals-1455044-supplementary.pdf]

Supplementary Table S1. Pre- and post-MLX treatment blood analytes in green and Kemp's ridley sea turtles*

Analytes	Pre-treatment n = 12	Post-treatment n = 12	p
**Plasma chemistry**			
AST U/L	484 (182, 817)	457 (212, 735)	0.73
Creatine kinase U/L	1516 (267, 13776)	1270 (381, 19484)	0.33
Albumin g/dL	1.0 (0.7, 1.7)	1.4 (0.7, 1.7)	0.17
Total Protein g/dL	3.5 (2.9, 4.1)	4.0 (3.2, 4.5)	< 0.01
Globulin g/dL	0.7 (0.4, 1.1)	0.8 (0.5, 1.0)	0.02
A/G Ratio	0.6 (0.3, 0.9)	0.6 (0.3, 0.7)	0.42
Urea Nitrogen mg/dL	86 (23, 103)	77 (18, 127)	0.76
Cholesterol mg/dL	185 (110, 386)	183 (141, 398)	0.06
Glucose mg/dL	110 (78, 124)	125 (93, 146)	< 0.01
Calcium mg/dL	6.4 (5.0, 7.9)	6.6 (5.7, 6.9)	0.96
Phosphorus mg/dL	9.7 (4.9, 13.1)	10 (5.50, 15.6)	0.05
Chloride mEq/L	118 (107, 125)	120 (107, 128)	0.05
Potassium mEq/L	4.1 (3.5, 5.2)	4.3 (3.2, 5.1)	0.14
Sodium mEq/L	150 (145, 164)	150 (145, 164)	0.19
Uric Acid mg/dL	0.8 (0.4, 1.5)	0.9 (0.6, 1.3)	0.81
Triglycerides mg/dL	189 (41, 487)	28 (50, 1244)	0.06
Total protein (refractometer) g/dL	4.0 (3.3, 5.2)	4.4 (3.6, 6.1)	0.07
**Protein electrophoresis**			
Albumin g/dL	1.00 (9.65, 1.72)	1.37 (0.66, 1.74)	0.17
Alpha-1 globulins g/dL	0.25 (0.14, 0.57)	0.24 (0.14, 0.58)	0.27
Alpha-2 globulins g/dL	0.49 (0.31, 0.83)	0.62 (0.36, 0.94)	< 0.01
Beta-globulins g/dL	0.79 (0.48, 1.18)	0.83 (0.57, 1.13)	0.30
Gamma-globulins g/dL	0.70 (0.44, 1.01)	0.75 (0.45, 1.04)	0.02
A/G ratio	0.57 (0.30, 0.88)	0.63 (0.29, 0.71)	0.42
**Hematology**			
WBC estimate/µl	12100 (8200, 14400)**	12700 (8200, 23900)**	0.41
Absolute Heterophils/µl	5400 (1400, 8200)**	7500 (740, 17000)**	0.24
Absolute immature heterophils/µl	0 (0, 0)**	0 (0, 160)**	ND
Absolute Lymphocytes/µl	4000 (2100, 5700)**	4200 (2800, 8100)**	0.70
Absolute Monocytes/µl	1600 (740, 1900)**	1600 (570, 2400)**	0.55
Absolute Eosinophils/µl	200 (0, 1000)**	100 (0, 640)**	0.09
Absolute Basophils/µl	80 (0, 1600)**	110 (0, 1100)**	0.29
Packed cell volume (%)	32 (28, 35)	30 (24, 34)	0.20
Polychromasia	0 (0, 3)	1 (0, 2)	0.37
Anisocytosis	(0, 2)	0 (0, 2)	0.75
Thrombocytes	adequate	adequate	

*Data are presented as median (minimum, maximum). **n = 11. NA = Not applicable. ND = Not determined

Supplementary Table S2. Pre- and post-MLX treatment blood analytes in green sea turtles*

Analytes	Pre-treatment n = 6	Post-treatment n = 6	p
**Plasma chemistry**			
AST U/L	281 (182, 352)	303 (212, 586)	0.56
Creatine kinase U/L	1258 (167, 11322)	964 (381, 19484)	0.99
Albumin g/dL	1.4 (1.1, 1.7)	1.4 (1.3, 1.7)	0.43
Total Protein g/dL	3.7 (2.9, 4.1)	4.1 (3.7, 4.4)	0.09
Globulin g/dL	0.8 (0.5, 1.0)	0.9 (0.7, 1.0)	0.21
A/G Ratio	0.7 (0.7, 0.9)	0.7 (0.6, 0.7)	0.20
Urea Nitrogen mg/dL	59 (23, 97)	33 (18, 80)	0.12
Cholesterol mg/dL	162 (110, 197)	161 (141, 272)	0.43
Glucose mg/dL	110 (78, 123)	120 (93, 130)	0.21
Calcium mg/dL	6.2 (5.0, 7.4)	6.5 (5.7, 6.8)	0.68
Phosphorus mg/dL	12.0 (4.9, 13.1)	11.8 (5.5, 15.6)	0.21
Chloride mEq/L	121 (107, 125)	117 (107, 128)	0.62
Potassium mEq/L	4.2 (4.0, 5.2)	4.7 (4.2, 5.1)	0.18
Sodium mEq/L	153 (145, 154)	155 (145, 164)	0.25
Uric Acid mg/dL	1.0 (0.7, 1.5)	1.0 (0.7, 1.3)	0.25
Triglycerides mg/dL	238 (77, 487)	191 (50, 1244)	0.84
Total protein (refractometer) g/dL	4.15 (3.30, 5.20)	4.30 (3.60, 6.10)	0.56
**Protein electrophoresis**			
Albumin g/dL	1.35 (1.08, 1.72)	1.43 (1.33, 1.74)	0.43
Alpha-1 globulins g/dL	0.22 (0.14, 0.27)	0.21 (0.14, 0.36)	0.87
Alpha-2 globulins g/dL	0.47 (0.31, 0.60)	0.62 (0.36, 0.72)	0.03
Beta-globulins g/dL	0.62 (0.48, 0.80)	0.78 (0.57, 0.84)	0.09
Gamma-globulins g/dL	0.79 (0.50, 1.01)	0.91 (0.70, 1.04)	0.21
A/G ratio	0.70 (0.66, 0.88)	0.67 (0.62, 0.71)	0.21
**Hematology**			
WBC estimate/µl	12100 (8400, 13600)**	9900 (8200, 11200)**	0.12
Absolute Heterophils/µl	5400 (1400, 7900)**	4000 (740, 6200)**	0.12
Absolute immat heterophils/µl	0 (0, 0)**	0 (0, 160)**	ND
Absolute Lymphocytes/µl	4400 (2100, 5700)**	4200 (3100, 8100)**	0.81
Absolute Monocytes/µl	1200 (840, 1800)**	1500 (570, 1600)**	0.99
Absolute Eosinophils/µl	130 (0, 820)**	0 (0, 100)**	0.25
Absolute Basophils/µl	360 (80, 1600)**	180 (0, 1100)**	0.12
Packed cell volume %	31 (28, 34)	31 (24, 34)	0.62
Polychromasia	0 (0, 3)**	1 (0, 2)**	ND
Anisocytosis	0 (0, 2)**	0 (0, 2)**	ND
Thrombocytes	adequate	adequate	

*Data are presented as median (minimum, maximum). **n = 5. NA = Not applicable. ND = Not determined

Supplementary Table S3. Pre- and post-MLX treatment blood analytes in Kemp's ridley sea turtles*

Analytes	Pre-treatment n = 6	Post-treatment n = 6	p
**Plasma chemistry**			
AST U/L	674 (616, 817)	634 (409, 735)	0.31
Creatine kinase U/L	1765 (908, 13776)	1353 (983, 1714)	0.21
Albumin g/dL	0.9 (0.7, 0.9)	0.90 (0.7, 1.7)	0.43
Total Protein g/dL	3.5 (3.1, 4.1)	3.9 (3.2, 4.5)	0.03
Globulin g/dL	0.6 (0.4, 0.8)	0.7 (0.5, 0.8)	0.03
A/G Ratio	0.4 (0.3, 0.5)	0.4 (0.3, 0.6)	0.99
Urea Nitrogen (BUN) mg/dL	98 (82, 103)	117 (68, 127)	0.09
Cholesterol mg/dL	194 (161, 386)	203 (181, 398)	0.06
Glucose mg/dL	113 (84, 124)	128 (106, 146)	0.06
Calcium mg/dL	6.8 (5.4, 7.9)	6.6 (6.0, 6.9)	0.68
Phosphorus mg/dL	9.0 (8.3, 9.8)	9.7 (8.3, 13.1)	0.31
Chloride mEq/L	115 (113, 123)	120 (115, 125)	0.03
Potassium mEq/L	3.7 (3.5, 4.2)	4.0 (3.2, 4.6)	0.56
Sodium mEq/L	149 (147, 151)	149, 154)	0.62
Uric Acid mg/dL	0.7 (0.4, 1.2)	0.9 (0.6, 1.0)	0.50
Triglycerides mg/dL	146 (41, 213)	334 (77, 553)	0.03
Total protein (refractometer) g/dL	4.0 (3.6, 5.0)	4.5 (3.8, 5.1)	0.03
**Protein electrophoresis**			
Albumin g/dL	0.85 (0.65, 0.93)	0.89 (0.66, 1.74)	0.43
Alpha-1 globulins g/dL	0.35 (0.23, 0.57)	0.38 (0.23, 0.58)	0.31
Alpha-2 globulins g/dL	0.49 (0.42, 0.83)	0.62 (0.50, 0.94)	0.03
Beta-globulins g/dL	1.00 (0.79, 1.18)	1.03 (0.71, 1.13)	0.56
Gamma-globulins g/dL	0.62 (0.44, 0.75)	0.70 (0.45, 0.81)	0.03
A/G ratio	0.41 (0.30, 0.48)	0.38 (0.29, 0.64)	0.99
**Hematology**			
WBC estimate/µl	12050 (8200, 14400)	15000 (12700, 23900)	0.03
Absolute Heterophils/µl	6200 (3500, 8200)	8650 (7500, 17000)	0.03
Absolute immat heterophils/µl	0 (0, 0)	0.00 (0.00, 0.00)	ND
Absolute Lymphocytes/µl	4000 (3400, 5300)	4100 (2800, 4600)	0.99
Absolute Monocytes/µl	1600 (740, 1900)	1750 (1500, 2400)	0.43
Absolute Eosinophils/µl	385 (0, 1000)	250 (0, 640)	0.31
Absolute Basophils/µl	0 (0, 110)	0 (0, 240)	ND
Packed cell volume %	32 (29, 35)	30 (26, 33)	0.21
Polychromasia	0 (0, 0)	0 (0, 0)	ND
Anisocytosis	0 (0, 0)	0 (0, 0)	ND
Thrombocytes	adequate	adequate	

*Data are presented as median (minimum, maximum). NA = Not applicable. ND = Not determined
